# The Development and Study of a New Silylated Polyurethane-Based Flexible Adhesive—Part 2: Joint Testing and Numerical Modelling

**DOI:** 10.3390/ma16217022

**Published:** 2023-11-03

**Authors:** Vasco C. M. B. Rodrigues, Eduardo A. S. Marques, Ricardo J. C. Carbas, Michael Youngberg, Anne Dussaud, Reza Beygi, Lucas F. M. da Silva

**Affiliations:** 1 Institute of Science and Innovation in Mechanical and Industrial Engineering (INEGI), Rua Dr. Roberto Frias, 4200-465 Porto, Portugal; up201806642@edu.fe.up.pt (V.C.M.B.R.); rcarbas@fe.up.pt (R.J.C.C.); 2 Department of Mechanical Engineering, Faculty of Engineering, University of Porto, 4200-465 Porto, Portugal; emarques@fe.up.pt (E.A.S.M.); lucas@fe.up.pt (L.F.M.d.S.); 3 Momentive Performance Materials Inc., 769 Old Saw Mill River Road, Tarrytown, NY 10591, USA; michael.youngberg@momentive.com (M.Y.); anne.dussaud@momentive.com (A.D.)

**Keywords:** SPUR, silylated polyurethane, CZM, flexible adhesive

## Abstract

The need for more sustainable adhesive formulations has presented the possibility of using silane-based adhesives in the automotive industry. In this work, a dual-cure two-component silylated polyurethane resin (SPUR) adhesive was tested in single-lap joints, to assess in-joint behaviour at room temperature under quasi-static conditions for aluminium substrates. The effect of two different overlap lengths, 25 and 50 mm, was also considered. A numerical model was built using cohesive zone modelling in finite element software, to reproduce the mechanical behaviour of the joint. The model was fed with data experimentally withdrawn from the first part of this paper. A triangular-shaped cohesive zone model (CZM) law was chosen as the adhesive behaviour was highly elastic and lacked yielding phenomena. The experimental results served as the base for the numerical validation, allowing accurate CZM parameters to be successfully determined.

## 1. Introduction

The advent of adhesive technology brought significant transformations in the realm of mechanical joining [1]. In particular, the traditional practices of riveting and fastening have been partially replaced by adhesive bonding or used in integrated methods in hybrid joints, which combine both approaches. The bonding of dissimilar materials in the automotive industry was solved by implying novel adhesive technologies, which have been increasing and developing to enhance structural components [2,3,4], along with their durability [5,6,7,8]. Flexible adhesives have emerged as vital components within the automotive industry [9,10,11,12,13], used in various applications such as sealants [14,15,16,17], structural adhesives [18,19,20], and semi-structural elements. These advancements align with the overarching goal of developing lightweight designs and embracing a more environmentally conscious philosophy [21,22]. Polyurethane adhesives are renowned for their mechanical flexibility, making them particularly suitable for bonding composite substrates [23,24,25,26,27,28]. This leads to a reduced risk of delamination, a common failure mode in composite bonding found, for instance, when using brittle and stiff adhesives such as epoxies [21]. This adhesive family exhibits a lower elastic modulus and comparatively lower tensile strength; however, it displays a significantly greater elongation capacity [29], generating lower transverse strains in composite adherends. Furthermore, these levels of elastic deformation positively contribute to achieving effective gaskets and sealing components, as well as exceptional damping, impact, and fatigue properties [30,31].

Silylated polyurethane resins (SPURs), which were used in the adhesive in this study, are a particular group of polyurethane materials, which contain silane groups in the polymer backbone [32,33,34]. Their curing relies on the hydrolysis/condensation of silane groups, which form siloxane bonds as cross-linking sites. They can be formulated as a one-part adhesive, which cures in the presence of a catalyst and moisture provided by the ambient atmosphere. Alternatively, the adhesive can be formulated in two stages for faster curing, with water provided in one of the stages [35]. Typically, these resins do not contain free isocyanates and tend to have a lower toxicity than their traditional polyurethane counterpart [36,37]. Silane-terminated polymers (STPs) have been used in construction due to their enhanced weathering stability, adhesion on multiple substrates, and satisfactory storage stability. In contrast, in the past two decades, STPs had limited use in adhesives for transportation, where polyurethanes or silicone adhesives have dominated in the applications of elastic bonding. However, due to the new environmental constraints, there is a renewed interest in hybrid resins for adhesive applications in electrical vehicles.

To support the wider industrial use of these adhesives, it is important to develop numerical models of the adhesive behaviour, properly validated through experimental tests. Thus, having established a suitable material model, the same adhesive can be modelled under different configurations, providing accurate failure prediction. Multiple methods of adhesive failure prediction have been analytically developed [21]. Currently, the finite element (FE) framework, combined with the cohesive zone method (CZM) as an add-in, has become the benchmark for the simulation of damage growth [38], based on traction separation laws. This methodology considers the stress–strain behaviour as well as fracture mechanics for failure prediction. It is considered superior to other modelling approaches such as the virtual crack closure technique (VCCT) or the linear elastic fracture mechanics (LEFM), which possess more limitations [39].

Cohesive zone modelling is suitable for adhesive joints since it can predict damage and failure with good accuracy in a predefined region. It simulates the elastic loading until a peak load is reached, followed by the damage process through crack initiation and propagation. The preferred damaging mode depends on the material behaviour or interface [38,40]. The predefined law shape takes into consideration the tensile, shear, and fracture toughness properties of the adhesive, establishing a failure and subsequent damage path [38]. Campilho et al. [38] compared the behaviour of different cohesive shape laws for brittle and ductile adhesives in single-lap joints (SLJs) with different overlap lengths tested under tension. The triangular shape law is generally suitable for brittle and stiff adhesives since it converges rapidly and does not consider large yielding. Furthermore, it was concluded that the shape law effect could be negligible in these adhesives. On the other hand, for ductile adhesives, the trapezoidal law is generally more accurate than the triangular and exponential shapes. Pisavadia et al. [41] studied a trapezoidal (trilinear) CZM shape and found that it was more suitable for the numerical validation of a polyurethane adhesive. The property identification methodology was previously performed in order to determine the mechanical adhesive properties and then establish an association between the real and cohesive values. For the triangular shape law, the elastic failure strength $\sigma_{f}$ and shear failure strength $\tau_{s}$ correspond to the tension and shear cohesive strengths $t_{n}^{0}$ and $t_{s}^{0}$, respectively. The area of the cohesive law shape is determined through the cohesive parameters $G_{n}^{c}$ and $G_{s}^{c}$, which represent the critical energy release rates in pure opening (mode I) and in-plane shear (mode II).

In this work, aluminium SLJs were tested with 25 mm and 50 mm adhesive overlap lengths, under quasi-static conditions. CZM elements in ABAQUS^®^ FE software 2021 (Dassault Systèmes, Suresnes, France) were used, following a triangular shape cohesive law to simulate the adhesive. The numerical model reproduced similar geometries to those used in the mechanical tests already performed, allowing for a direct comparison of the experimental and numerical results. In previous works, trilinear CZM shape laws have been used in polyurethane adhesives [21,30,38,40,41]; however, since the experimental results of this work showed no yielding point in both tensile and shear tests, a simpler triangular shape cohesive law was found to be more effective. Thus, the aim is the numerical simulation of the behaviour of a new SPUR formulation.

## 2. Materials

A two-component (2k) SPUR adhesive was mechanically characterised in Part 1 of this paper using four different tests, namely the bulk test, following the NF T 76-142 French standard [42]; the thick adherend shear test (TAST) according to the ISO 11003-2 standard [43]; the double-cantilever beam test; and a mixed-mode configuration test [44]. Table 1 summarises the mechanical properties of the 2k adhesive. The critical energy release rate in mode II was estimated from the mixed-mode fracture envelope output. All fracture characterisation tests employed the compliance-based beam method (CBBM) [44,45,46,47]. Poisson’s ratio was determined using the digital image correlation (DIC) in the tensile bulk specimens, where a shear modulus value of 3.59 MPa was found, considering the elastic and shear modulus relation for isotropic materials [48]. The shear moduli presented in Table 1 correspond to the values obtained from the TAST specimens, accounting for the different strain rates and different boundary conditions of both tensile and TAST experiments obtained in the first part of this paper.

## 3. Experimental Procedures

### Aluminium SLJ

**Figure 1:**
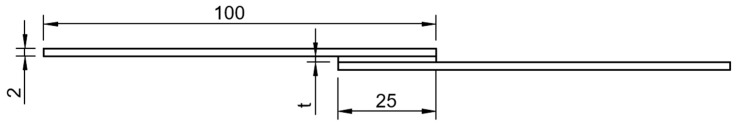
Aluminium SLJ geometry with a 25 mm overlap length.

Anodised AL6060-T6 aluminium alloy substrates were used in this work to build the SLJs. The adherend elastic properties of this material are displayed in Table 2, and the general geometry of the aluminium joints manufactured is illustrated in Figure 1, where the adhesive layer was set to be 0.5 mm thick with an overlap length of 25 or 50 mm.

**Figure 2:**
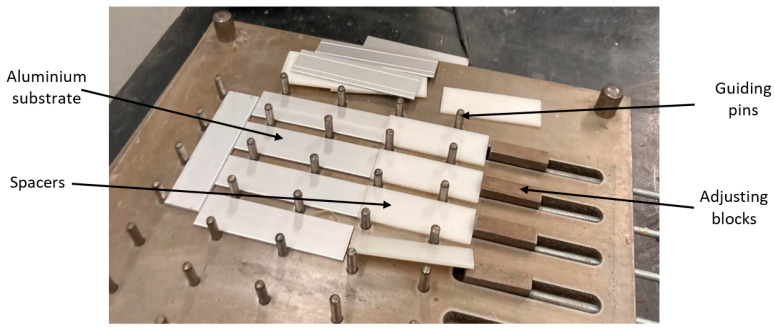
Mould for the SLJs.

SLJs were mounted in a steel mould [49], previously cleaned and coated with release agent film (Figure 2). To control the specimen geometry, 3D-printed polymeric spacers were used, coated with release agent film to facilitate removal after adhesive curing. Since the aluminium substrates were anodised, the surface was simply degreased with isopropyl alcohol (IPA) before adhesive application. Figure 1 shows an image of the stage in which the aluminium substrate SLJs with 25 mm overlaps were prepared.

Following assembly, a set of lap shear test specimens was placed into a thermal chamber with weights on top, to guarantee mould closure, and maintained at 50 °C for 24 h. After curing, the rig was opened, the joints were retrieved, and the extra adhesive was removed from the overlap area. Alignment tabs of 25 × 25 mm were bonded to the end of each adherend to reduce load misalignment and peel effect when testing [21].

## 4. Experimental Results

**Figure 3:**
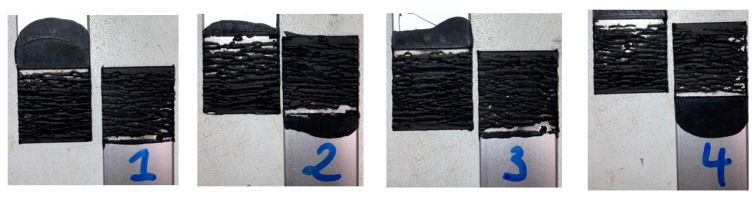
Modes of failure for all aluminium SLJs of 25 mm overlap specimens.

**Figure 4:**
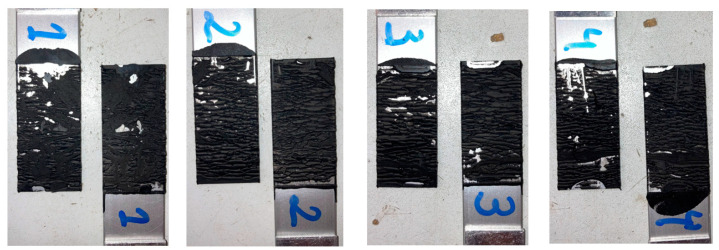
Modes of failure for all aluminium SLJs of 50 mm overlap specimens.

The SLJs were tested at a quasi-static constant crosshead rate of 1 mm/min at room temperature (RT) in an Instron 3832 (Norwood, MA, USA) quasi-static machine equipped with a 30 kN load cell. Figure 3 and Figure 4 display the modes of failure for both 25 and 50 mm overlap lengths, respectively.

**Figure 5:**
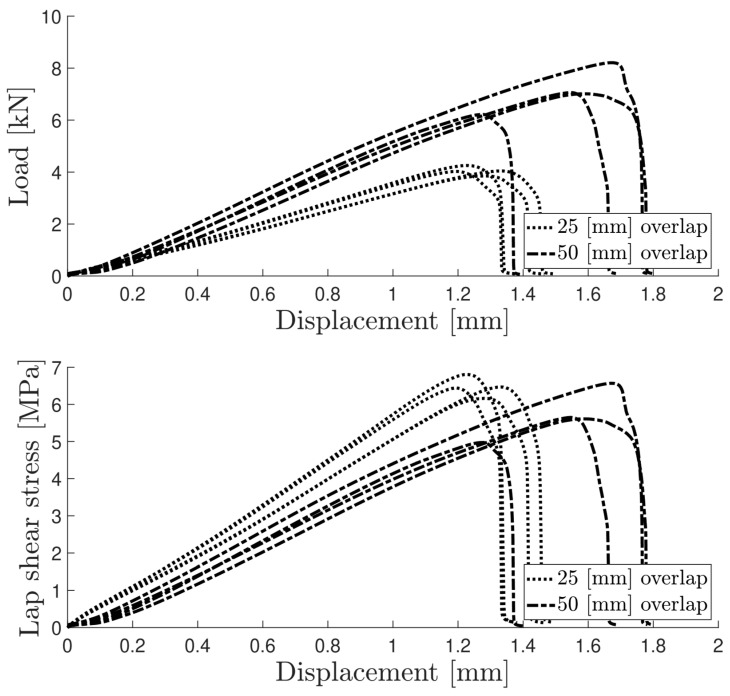
*P*–*δ* and lap shear stress curves for both aluminium SLJ overlap lengths.

Cohesive failure occurred for all specimens with both overlap lengths. The failure did not originate as a single crack along the overlap edges but led instead to the cracking of multiple sites throughout the thickness. Thus, the two-component formulation showed good adhesion with the anodised aluminium substrates. Figure 5 shows the load–displacement or *P*–*δ* curves, as well as the lap shear stress evolution, for both overlap lengths. The lap shear stress values, when testing under different bonding areas, generally remained very close, as a result of the large flexibility of the adhesive.

**Figure 6:**
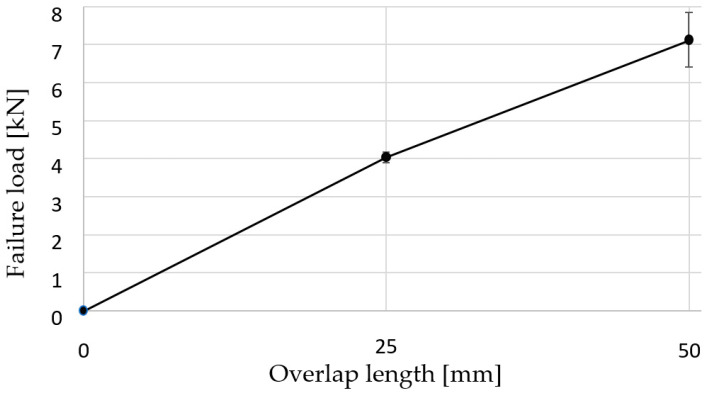
Evolution of the failure load with the overlap length.

Table 3 summarises the experimental results of the lap shear tests. The lap shear stress values did not vary significantly between the two overlap lengths (Figure 6).

## 5. Numerical Modelling

### 5.1. Numerical Details

ABAQUS^®^ FE software 2021 was used to perform a numerical analysis in which a triangular shape CZM law was applied to cohesive adhesive layers. The adherents used in the TAST, double-cantilever beam (DCB) test, and SLJ test procedures were modelled following a linear elastic approach, using ABAQUS^®^ CPS4R plane-strain elements with reduced integration and hourglass control. The adhesive layer was modelled using COH2D four-node cohesive elements, chosen due to their compatibility with the adherend selected elements. A mesh refinement procedure was performed along the adhesive overlap lengths using a double-bias parameter, allowing for an increase in mesh density at the overlap edges. Unlike the bidimensional models, the three-dimensional bulk specimen used eight-node hexahedron (C3D8R) 3D elements with reduced integration and hourglass control.

**Figure 7:**
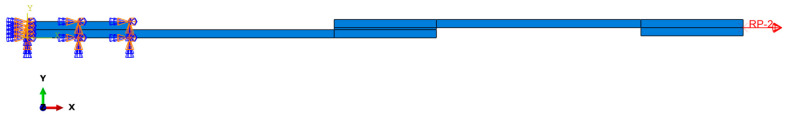
Boundary conditions of the SLJ model.

The chosen boundary conditions simulated the fixtures used in the experimental tests. The bottom edge of the bulk specimen was fixed, and a displacement load was applied to the opposite edge. This design was applied to both TAST and SLJ models, although in the latter alignment, tabs were also added to the model geometry, as displayed in Figure 7.

**Figure 8:**
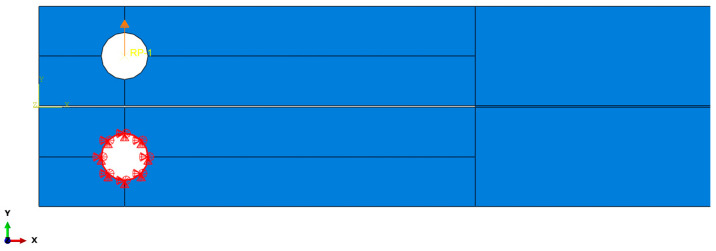
Boundary conditions for the DCB specimen model.

The lower hole of the DCB specimen was pinned, allowing for rotation along the normal axis to the surface, with an induced displacement along the *y* axis for the upper hole, as illustrated in Figure 8. Although a more dense and refined mesh over the total bonded area ought to provide a smoother *P*–*δ* curve, a single-bias mesh from the crack tip until the end of the specimen was found to provide similar results with less computational time.

The elastic behaviour of cohesive zone models was simply defined as a linear correlation between the maximum strength and the modulus: This applied to both tensile (*n*) and shear (*s*) scenarios. The cohesive traction is determined as the stiffness matrix **K** multiplied by the respective strains. The stiffness matrix contains the tensile and shear stiffness parameters, where *K_nn_* = *E* and *K_ss_* = *G*. Since no tensile–shear mix was performed, *K_ns_* = *K_sn_*, and it was assumed to be null [38].

(1) $$t=K\varepsilon \;\Leftrightarrow \left\{\begin{array}{c} t_{n}\\ t_{s} \end{array}\right\}=\left[\begin{array}{cc} K_{nn} & K_{ns}\\ K_{sn} & K_{ss} \end{array}\right]\left\{\begin{array}{c} \varepsilon_{n}\\ \varepsilon_{s} \end{array}\right\}$$

The damage criterion selected was a quadratic nominal stress formulation, which is described using the following equation:

(2) $${\left(\frac{t_{n}}{t_{n}^{0}}\right)}^{2}+{\left(\frac{t_{s}}{t_{s}^{0}}\right)}^{2}=1$$

The softening phenomenon occurs after the load peak is reached. For a triangular shape law, the traction term is defined as follows:

(3) $$t_{n}=\left(1-d_{n}\right)t_{n}^{u}$$

(4) $$t_{s}=\left(1-d_{s}\right)t_{s}^{u}$$

where $t_{n}^{u}$ and $t_{s}^{u}$ are the instantaneous cohesive traction. The damage variables *d_n_* and *d_s_* refer to tension (normal) and shear, respectively, and following the triangular shape law, they are expressed as follows [38]:

(5) $$d_{n,s}=\frac{\delta_{n,s}^{f}\left(\delta_{n,s}-\delta_{n,s}^{0}\right)}{\delta_{n,s}\left(\delta_{n,s}^{f}-\delta_{n,s}^{0}\right)}$$

which is valid for both tension and shear components.

**Figure 9:**
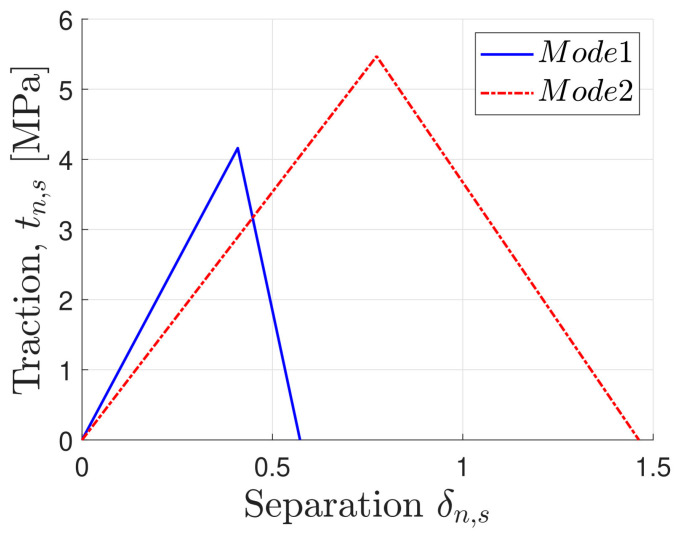
Triangular shape cohesive law chosen.

The definition of the damage criterion for traction–separation laws is based on an energetic approach, where the critical energy release rate values for both mode I and mode II are considered [21]. Thus, it is possible to extrapolate a mixed-mode fracture energy relationship by combining the individual laws into a unified mixed-law formulation. In one approach, the power law is used, which is described as follows:

(6) $${\left(\frac{G_{I}}{G_{I_{c}}}\right)}^{\alpha }+{\left(\frac{G_{II}}{G_{II_{c}}}\right)}^{\alpha }=1$$

where the value for the *α* parameter chosen was equal to one, thus resulting in a linear relationship. The estimated values were interpolated to plot the triangular shape laws in Figure 9, used in the cohesive elements [40].

The shear modulus value used in the model was the one determined using the thick adherent shear test.

### 5.2. Numerical Validation

**Figure 10:**
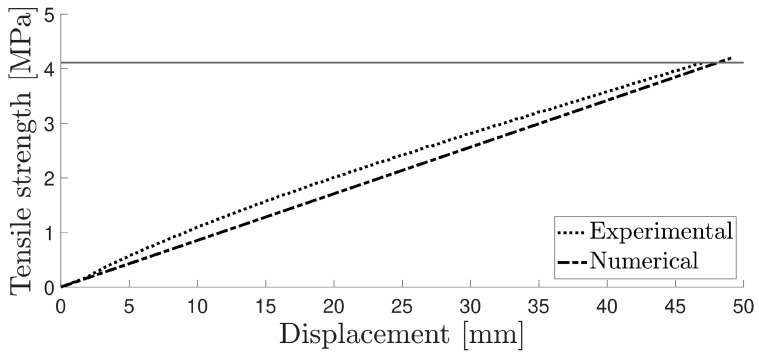
Numerical and experimental curve for the tensile test.

The tensile behaviour was assumed to follow a linear elastic behaviour, as the material tensile properties did not present a yield point followed by a plateau. Figure 10 illustrates the tensile strength–displacement curve for a typical experimental run with the numerical model.

**Figure 11:**
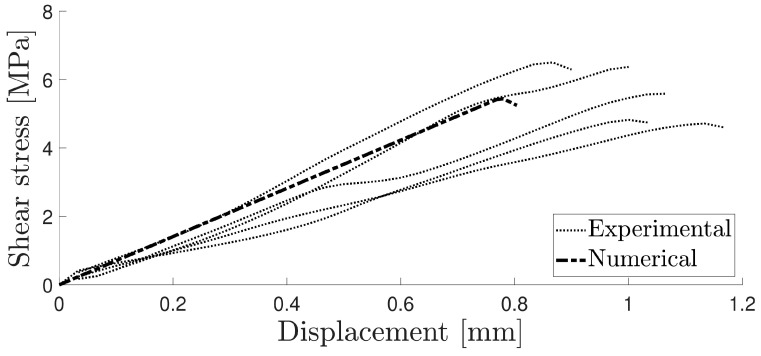
Numerical and experimental curve for the TAST.

In the shear test, significant variations were observed in the experimental curves due to the low adhesive stiffness. A satisfactory correlation was found by employing the same triangular shape cohesive law, as shown in Figure 11.

In the experimental testing process of the SPUR DCB specimen, half of the samples underwent a precracking process. The DCB specimens were subjected to the same quasi-static rate, but the test was stopped when the *P*–*δ* curve reached a maximum, nucleating a crack.

**Figure 12:**
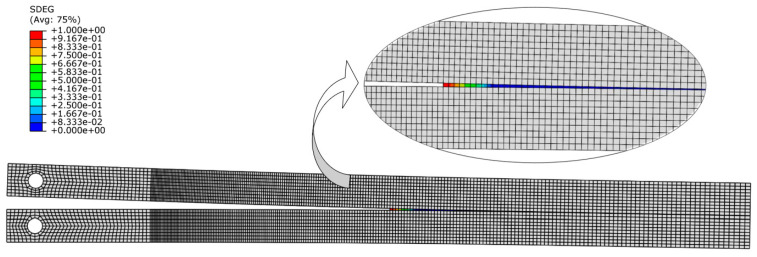
Damage evolution on the DCB cohesive layer.

**Figure 13:**
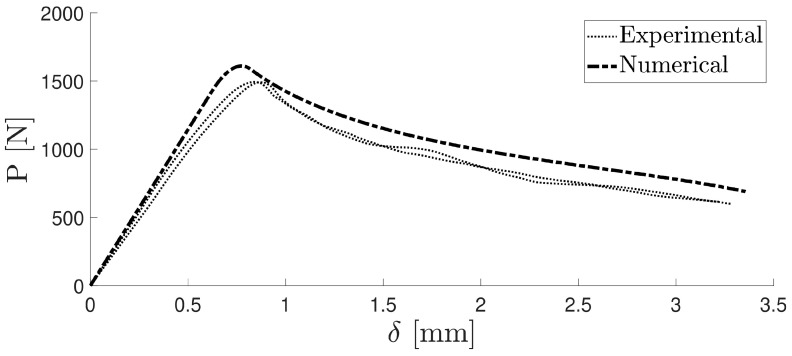
Numerical and experimental *P*–*δ* curve.

**Figure 14:**
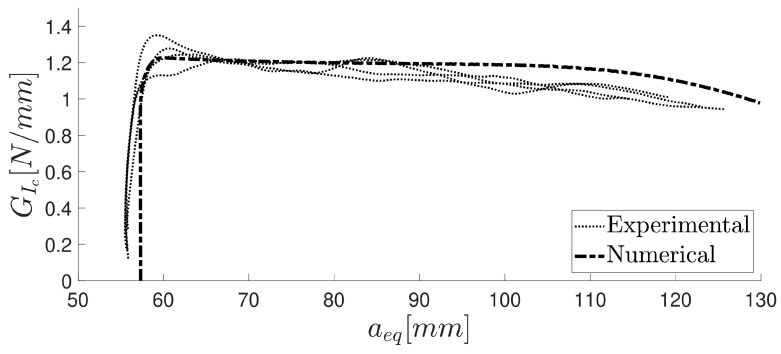
R curve computed using the CBBM for the numerical and experimental data modulus values calculated using the thick adherend shear test and another one obtained by extrapolating the value from the estimation of the Poisson’s ratio from the tensile test, using the outputs obtained with DIC software (2019). The higher shear modulus value provided a more accurate result over the bulk extrapolated value.

Then, those specimens were tested after the precracking stage was completed. The previous study showed that the measurement of the critical energy release rate *G_I__c_* does not depend on whether the specimen undergoes precracking. The results of the numerical study were compared with the experimental results for both the precracked and non-precracked specimens. Figure 12 illustrates the damage evolution value of the cohesive layer at a particular opening stage of the specimen, and Figure 13 displays the *P*–*δ* curve for the numerical simulation and the precracked specimens. The R curves obtained with the compliance beam-based method (CBBM) [45], for both numerical and experimental conditions, are displayed in Figure 14.

**Figure 15:**
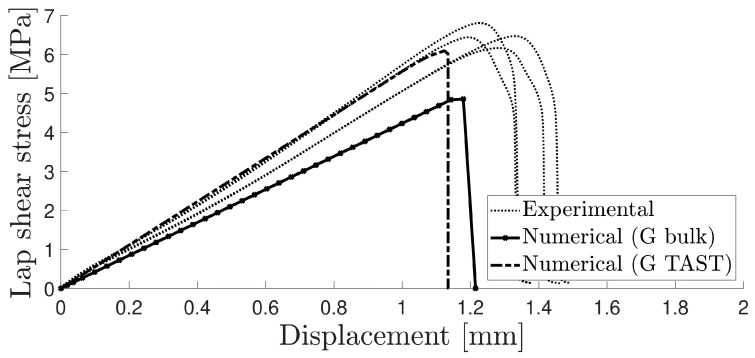
Lap shear strength–displacement curve for both numerical and experimental results of aluminium SLJs with 25 mm overlaps.

The analysed aluminium SLJs exhibited consistent testing results, with cohesive failure. Figure 15 shows the experimental data with two numerical curves: one using the thick adherend shear test and the other using the value obtained from the estimation of the Poisson’s ratio from the tensile test, performed with the DIC software (2019).

## 6. Conclusions

In this paper, a new two-component SPUR adhesive with epoxy resin was tested for SLJs and numerically modelled using a triangular cohesive shape law in the ABAQUS^®^ FE software 2021. Numerical models for the tensile test, TAST, DCB test, and SLJ test were also built in ABAQUS^®^ FE software 2021, where the adhesive properties were validated, allowing us to obtain a numerical curve in agreement with the experimental data. The bulk specimen model used a linear elastic approach with which the maximum tensile stress helped to define the failure criterion. On the other hand, in the TAST, DCB test, and SLJ test, the adhesive cohesive properties were considered, and the shear modulus value was determined in the TAST. In fact, the influence of the tensile modulus was negligible for all three models, since the adhesive layer was much more constrained than in a tensile test specimen. The following remarks summarise the key outcomes of this study:

- SLJs were manufactured using anodised aluminium substrates. The adhesive showed good adhesion to the aluminium substrates, providing a lap shear strength of 6.47 MPa in joints with a 25 mm overlap length.
- The triangular shape CZM law, commonly found to be suitable for brittle and stiff adhesives, was nonetheless able to simulate the elastic behaviour of the 2k SPUR with no yielding point. The material properties and the CZM law determined from this model successfully simulated the in-joint behaviour of the adhesive under quasi-static conditions.

## Figures and Tables

**Table 1:** The mechanical properties of the 2k SPUR.

Property	Units	2k SPUR
Young’s modulus, *E*	(MPa)	10.17 ± 0.96
Poisson’s ratio, *ν*	(-)	0.418 ± 0.009
Tensile failure strength, *σ_f_*	(MPa)	4.16 ± 0.21
Tensile failure strain, *ε_f_*	(%)	41.1 ± 5.8
Shear modulus, G	(MPa)	7.07 ± 1.53
Shear failure strength, *τ_f_*	(MPa)	5.47 ± 0.74
Shear failure strain, *γ_f_*	(%)	84.7 ± 11.5
Toughness in mode I, *G_Ic_*	(N/mm)	1.191 ± 0.055
Toughness in mode II, *G_IIc_*	(N/mm)	4

**Table 2:** Mechanical properties for the anodised aluminium substrates.

Elastic Modulus, *E*	Poisson’s Ratio, *ν*
(GPa)	(-)
69	0.33

**Table 3:** The test results of aluminium SLJs.

Overlap	Maximum Loading, *F_max_*	Lap Shear Stress, *σ_lss_*	Extension at *F_max_*
(mm)	(kN)	(MPa)	(mm)
25	4.04 ± 0.14	6.47 ± 0.23	1.230 ± 0.061
50	7.13 ± 0.71	5.70 ± 0.57	1.521 ± 0.147

## Data Availability

Not applicable.
